# Transfacet Oblique Lateral Lumbar Interbody Fusion: Technical Description and Early Results

**DOI:** 10.7759/cureus.26533

**Published:** 2022-07-03

**Authors:** Hamid Abbasi, Nicholas R Storlie, Kessiena L Aya

**Affiliations:** 1 Ambulatory Surgical Clinic, Tristate Brain and Spine Institute, Alexandria, USA; 2 Neurosurgery, Inspired Spine Health, Minneapolis, USA; 3 Spine Surgery, Fairview Ridges, Burnsville, USA; 4 Orthopedic Surgery, University of Colorado, Denver, USA; 5 Orthopedic Surgery, University of Texas Medical Branch at Galveston, Galveston, USA

**Keywords:** l5-s1 level, spinal fusion, spine, ollif, lumbar interbody fusion, oblique lateral lumbar interbody fusion, minimally invasive surgery

## Abstract

Introduction

The oblique lateral lumbar interbody fusion (OLLIF) is a relatively new method of lumbar interbody fusion (LIF) that utilizes a trans-Kambin approach to the disc space. The OLLIF can be performed from T12-S1 in the majority of cases but is occasionally obstructed at the L5-S1 level by osteophytes, an overgrown facet joint and/or prominent sacral ala. Transfacet OLLIF (TF-OLLIF) is a novel method for LIF in which the disc space is accessed by drilling through hypertrophic facets with an OLLIF approach. We provide a proof-of-concept report on the TF-OLLIF surgical technique and report the clinical and perioperative outcomes for the first 29 patients who underwent this procedure.

Methods

This is a retrospective single surgeon cohort study of 29 patients with lumbar spinal stenosis (LSS) who underwent TF-OLLIF procedures between 8/2018 and 1/2021. The primary outcome was a change in the Oswestry Disability Index (ODI) one year after surgery. Secondary outcomes were surgery time, blood loss, hospital stay, and complications. The TF-OLLIF was performed using the approach and instrumentation of OLLIF. When osseous hypertrophy is reached during the approach, an 8 mm drill is used to drill through the obstructing bone with continuous neuromonitoring. Discectomy and interbody placement are performed with subsequent posterior pedicle screw fixation.

Results

ODI improved from 49% pre-op to 31% at one-year follow-up. Estimated blood loss ranged from 97.6±93.3 ml for one level TF-OLLIF to 146.2±60.3 ml for a 3+ level TF-OLLIF. Operative time ranged from 57.4±19.5 minutes for a one-level TF-OLLIF to 102.9±27.8 minutes for a 3+ level TF-OLLIF. The average length of hospital stay (LOS) was 0.4±0.8 days for one-level TF-OLLIF and 1.6±1.9 days for 3+ level TF-OLLIF. Complications included five cases of nerve root irritation immediately postoperatively, with three of these patients still reporting mild L5 distribution numbness at the last follow-up, which was not clinically limiting.

Conclusion

The first 29 cases of TF-OLLIF demonstrated that it is a safe method of interbody fusion that yields good clinical results. This is an important development for practitioners of OLLIF as it enables interbody placement with OLLIF instruments and approach even for challenging L5-S1 levels without compromising surgical outcomes.

## Introduction

Low back pain is a condition that affects up to 80% of US adults at some point in their lives [1]. While a significant number of these cases may be successfully treated conservatively, a number develop into chronic conditions which cause significant morbidity. One common source of lower back and radicular leg pain in our aging population is degenerative lumbar spinal stenosis (LSS). This is defined by compression of the spinal cord or nerve roots due to a reduction in the size of the central canal, lateral recesses, or neuroforamina [2]. Numerous anatomical processes seen in normal aging can be responsible for LSS. These include degenerative disc disease resulting in loss of intervertebral disc height, disc protrusions/herniation, and osteophyte or ligamentous hypertrophy [3]. Degenerative spondylolisthesis and scoliosis can also result in LSS through osteogenic narrowing of the central canal, lateral recesses, or neuroforamina [4].

When conservative therapies fail, surgical decompression is the standard of care in appropriate patients. However, decompression removes anatomic structures and may result in spinal instability that can worsen the underlying pathology [5]. Lumbar interbody fusions (LIF) are a common surgical intervention for LSS, which can restore stability to destabilized segments while providing an indirect/physiologic decompression of neural structures [6]. Although these treatments have been traditionally associated with substantial iatrogenic complications, the emergence of minimally invasive methods of fusion has reduced the risks involved in lumbar fusion [7].

We have previously described a relatively new approach to LIF, oblique lateral lumbar interbody fusion (OLLIF) [8]. This technique approaches the anterior column through Kambin's triangle, an electrophysiologically silent window of space between the exiting nerve root, superior border of the inferior vertebra, and superior articulating process of the inferior vertebra. This procedure has been shown to effectively provide physiologic decompression of neural structures in patients with LSS while being associated with low operation time, length of hospital stay (LOS), and estimated blood loss (EBL) [9,10].

Although the OLLIF can be safely performed from T12-S1, the L5-S1 level can occasionally be technically challenging due to the anatomic obstruction of Kambin's triangle. In patients with a prominent sacral ala or osteophytic growth surrounding a pathological facet joint, safely achieving the normal approach through Kambin's triangle can be infeasible. One proposed solution is a trans-iliac approach which inserts an interbody through an opening created in the iliac crest, but this has many technical limitations and little adoption by surgeons. Furthermore, given the challenges associated with L5-S1 lumbar fusion in OLLIF and other minimally invasive surgeries (MIS) such as anterior lumbar interbody fusion (ALIF), direct lateral interbody fusion (DLIF), and oblique lumbar interbody fusion-anterior to psoas (OLIF-ATP), developing safe approaches to this level is imperative [11-13].

We report a novel technique of L5-S1 LIF, which approaches the L5-S1 disc space with the same approach as OLLIF but utilizes drills to make space through the lateral aspect of a hypertrophied facet to allow passage of an interbody cage. This surgery, the transfacet OLLIF (TF-OLLIF), allows for an OLLIF approach in cases where this was previously impossible. This proof-of-concept study was conducted to determine whether the approach was technically feasible with a comparable safety profile and clinical outcomes to other techniques.

## Materials and methods

Study design

This study is a retrospective cohort study of 29 patients who underwent TF-OLLIF procedures between 9/2018 and 01/2021. Many of the patients included in this study underwent multi-level fusions, of which only one level was performed using the TF-OLLIF technique. For the purposes of this study, those procedures will be referred to as TF-OLLIF. Procedures were performed by a single surgeon in five hospitals and surgery centers in Minnesota, US. Institutional review board (IRB) exemption was granted by Pearl Pathways IRB. Inclusion criteria for this study were patients >18 years who underwent the OLLIF procedure where one or more levels were performed using the TF-OLLIF procedure. Indications for surgery included spondylolisthesis, degenerative disk disease, and disk herniation. All patients underwent preoperative imaging, including magnetic resonance imaging, an X-ray of the lumbar spine with flexion and extension, and, in many cases, a computed tomography (CT) scan and, in many cases, a discogram. Stenosis was diagnosed based on the presence of symptoms and imaging findings at the discretion of the principal investigator. Exclusion criteria were osteogenic spinal canal stenosis, grade II or greater spondylolisthesis, or other gross deformities. All patients underwent at least six months of conservative therapy, including physical therapy, therapeutic injections, bracing and behavioral modifications, before being considered candidates for surgery.

The TF-OLLIF procedure

The TF-OLLIF procedure is a variation of the OLLIF procedure we have previously described [8]. In brief, the patient is positioned on the operating table in the prone position, and biplanar fluoroscopy is set up. Prior to navigation to the disk, a Jamshidi needle is placed into an L5 pedicle, and bone marrow is drawn into a 10 CC syringe through the Jamshidi and applied to the bone graft. A Kirschner wire (K-wire) is placed through the Jamshidi for later pedicle screw placement, and the Jamshidi is removed.

**Figure 1:**
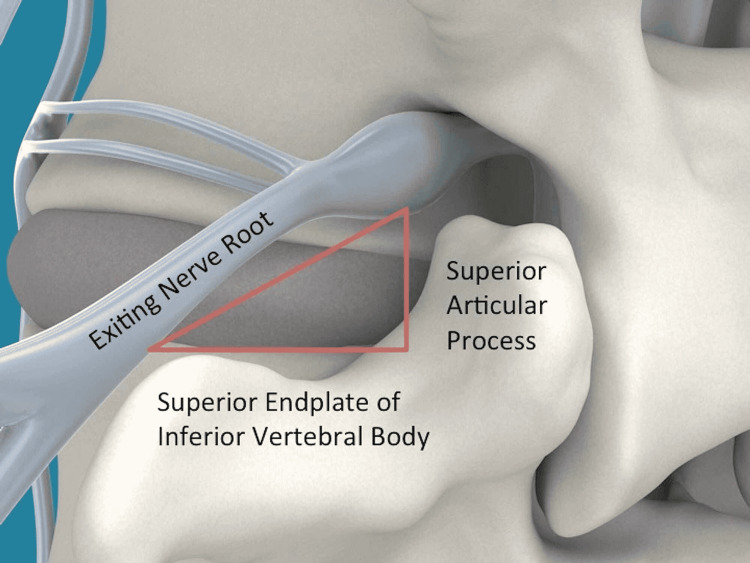
Depiction of Kambin's triangle

The disk is approached at a 45° angle to the vertical plane so that the instrumentation can pass through Kambin's triangle (Figure 1).

**Figure 2:**
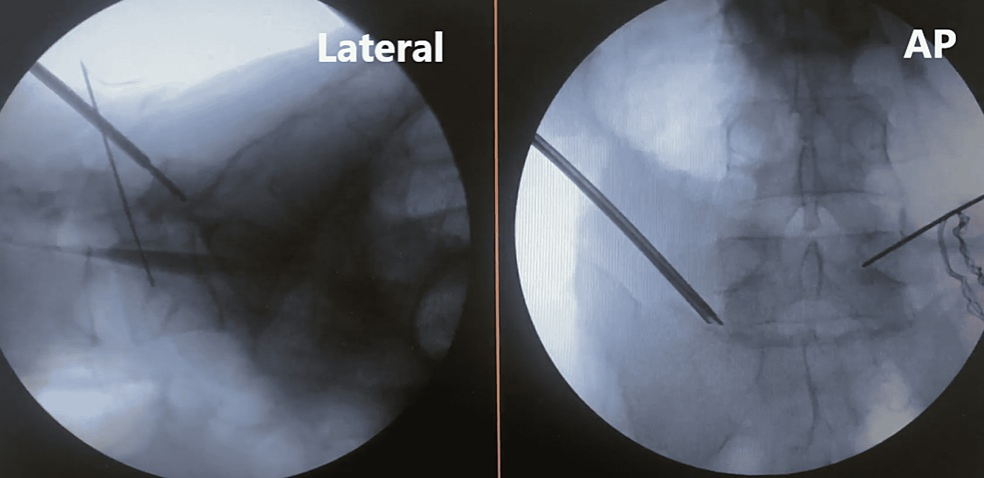
K-wire placement on the hypertrophied facet Lateral (left) and anterior/posterior (AP, right) of K-wire placement following the docking of the blunt probe on the hypertrophied facet.

The disk is first approached with a blunt neuromonitoring probe with the same trajectory as the OLLIF. Although the need for the TF-OLLIF technique may be anticipated based on preoperative radiological findings of hypertrophic facet joints, the use of the technique is an intraoperative decision made when the normal OLLIF approach is obstructed. The OLLIF approach is attempted twice with stimulation of 4 mA, and when a neurophysiologic silent area is not found, then TF-OLLIF is performed as a rescue technique. This is because, in our experience, making three or more attempted passes through Kambin's triangle increases the rate of nerve root irritation. When an osteophytic structure is reached with the desired trajectory, and the probe is stimulated up to our safety threshold of 10 mA, the probe sleeve is docked, and a K-wire is placed through the probe sleeve into the bone (Figure 2).

**Figure 3:**
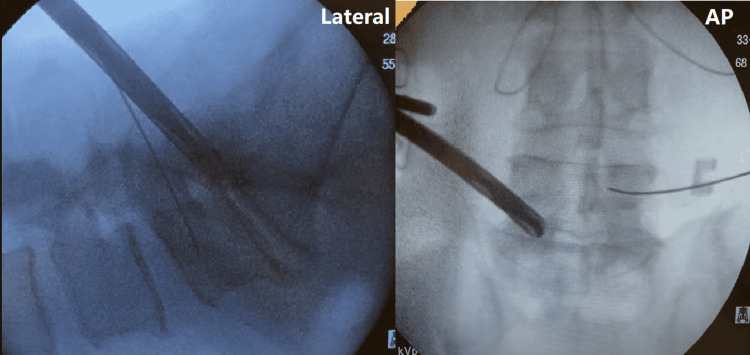
Approach through the hypertrophied facet is drilled through the access portal Lateral (left) and anterior/posterior (AP, right) fluoroscopy of the drill being used.

The probe sleeve is removed, and a blunt dilator is inserted over the K-wire, followed by a 10 mm access portal which is cored onto the bone. The dilator and K-wire are removed, and a drill is placed through the access portal to remove the obstructing bone (Figure 3).

**Figure 4:**
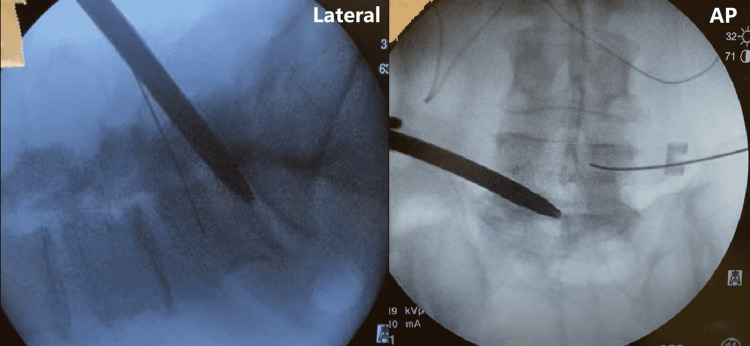
The dilator is entered into the disc space Lateral (left) and anterior/posterior (AP, right) fluoroscopy of the access portal being entered into the disc space, expanding the disc space and allowing the entrance of the access portal.

We alternated drilling and advancing the access portal until the disc space was reached. A neuromonitoring clip is attached to the access portal and continuously stimulated at 10 mA while the drill is being advanced to ensure we maintain a proper distance from neural structures. After the access portal has reached the vertebral body, the K-wire is placed through the access portal into the disc space. The dilator is reinserted over the K-wire into the disc space to expand the disc space for subsequent entry of the access portal (Figure 4). 

**Figure 5:**
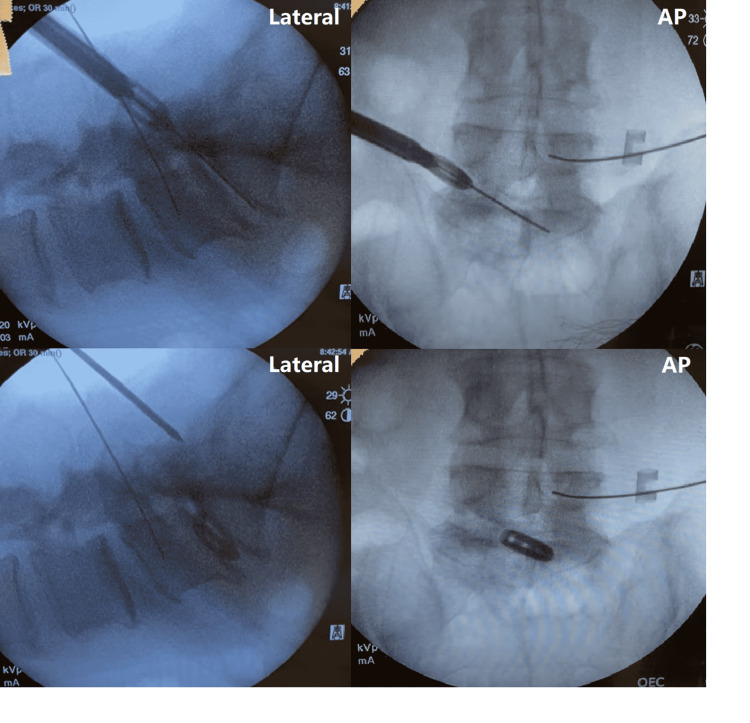
The cage is placed after discectomy and placement of the K-wire The top left and top right images are lateral and anterior/posterior (AP) fluoroscopy, respectively, of the interbody as it reaches the disc space. The bottom left and right images are lateral and AP fluoroscopy of the final interbody placement.

Discectomy is then performed through the 10 mm access portal consistent with the previously reported OLLIF procedure. The cage is inserted under continued electrophysiological monitoring and fluoroscopy (Figure 5). 

**Figure 6:**
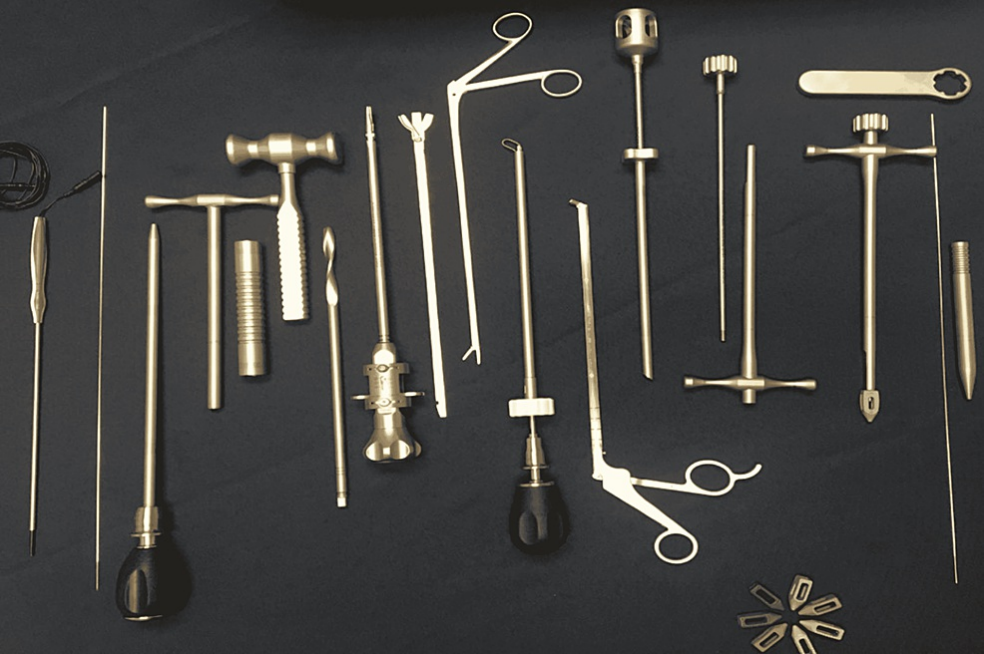
Instruments and implants used for interbody placement in transfacet OLLIF Instruments from left to right: neuromonitoring probe, K-wire, dilator, access portal, impactor, mallet, 8mm drill, paddle shaper and blade, pituitary rongeur, flexible curette, articulating rake, graft delivery tube and graft tamp,  inserter set (separated and assembled), and wrench. Interbodies are seen on the bottom right. OLLIF - oblique lateral lumbar interbody fusion

The interbody placement was accomplished using either the Zeus-O system from Amendia, Inc. (Marietta, US) or the OLLIF system from Advanced Research Medical (Burnsville, US), as shown in Figure 6. 

Lateral and anterior/posterior (AP) fluoroscopy of the procedure can be seen in Video 1 and Video 2, respectively.

**Video 1:** Lateral fluoroscopic view of the TF-OLLIF procedure TF-OLLIF - transfacet oblique lateral lumbar interbody fusion

**Video 2:** AP fluoroscopic view of the TF-OLLIF procedure AP - anterior/posterior; TF-OLLIF - transfacet oblique lateral lumbar interbody fusion

Finally, interbody placement is complemented with posterior percutaneous pedicle screw fixation to enable bilateral posterolateral fusion [14]. Posterior instrumentation was performed using the Pathloc-L MIS system from LnK Biomed (Seoul, South Korea).

Outcome measures and analysis

Immediately following surgery, the skin-to-skin operative time, EBL, and fluoroscopy time were recorded and entered into a custom database. Length of hospital stay was recorded in this database following discharge. EBL was calculated by measuring the weight of blood-saturated sponges and subtracting the dry weight of the sponges. Wound infections, bleeding, and other complications were recorded in the database following surgery and at follow-up.

Patients underwent a physical examination and completed a modified Oswestry Disability Index (ODI) 16 before surgery and at the one-year follow-up, which was defined as having taken place at least 300 days after surgery to allow for flexible patient scheduling. Nerve deficits were classified as nerve root irritation if the patient exhibited dysesthesia, paresthesias, or motor weakness of low clinical significance (4/5 or better). Deficits were classified as neuropraxia if the patient exhibited weakness of 3/5 or worse. Deficits were only categorized as complications if they were not present before surgery, appeared immediately after, and corresponded to the levels operated on during surgery. Radiculopathy was assessed both at the initial follow-up visit, which occurred within 90 days after surgery, and at the one-year follow-up, which took place at least 300 days after surgery. Following data collection, patient data was exported for analysis and visualization in R 3.4 (R Foundation for Statistical Computing, Vienna, Austria).

## Results

Study population demographic information and indications for surgery can be seen in Table 1.

**Table 1:** Study group characteristics and indications for surgery

Study group characteristics	Value count (%) or mean (SD)
n	29
BMI, mean (SD)	31.7 (6.7)
Age, mean (SD)	57.8 (13.0)
Degenerative or herniated disk, n (%)	24 (82.8)
Scoliosis, n (%)	6 (20.7)
Spondylolisthesis, n (%)	11 (37.9)
Stenosis, n (%)	10 (34.5)

The perioperative outcomes are listed in Table 2. Of the 29 patients included in this study, seven underwent one-level TF-OLLIFs, 14 underwent two-level fusions, where TF-OLLIF was performed at the L5-S1 level, and eight underwent 3+ level fusions where TF-OLLIF was performed at the L5-S1 level. The longest fusions performed were a five-level L1-S1 fusion and a four-level L2-S1 fusion. EBL, fluoroscopy time and LOS varied depending on the number of levels performed. EBL ranged from 97.6±93.3 ml for one-level TF-OLLIF to 146.2±60.3 ml for a 3+ level TF-OLLIF. Operative time ranged from 57.4±19.5 minutes for a one-level TF-OLLIF to 102.9±27.8 minutes for a 3+ level TF-OLLIF. Fluoroscopy ranged from 189.3±89.6 seconds for a one-level TF-OLLIF to 457.1±236.7 seconds for a 3+ level TF-OLLIF. Average LOS was 0.4±0.8 days for one-level TF-OLLIF and 1.6±1.9 days for 3+ level TF-OLLIF.

**Table 2:** Perioperative outcomes and demographic information by level

Perioperative Outcomes	Number of levels			
	1	2	3+	Overall
Number of cases	7	14	8	29
BMI, mean (SD)	28.1 (5.8)	33.4 (5.2)	31.8 (9.2)	31.7 (6.7)
Age, mean (SD)	52.7 (12.6)	57.2 (15.2)	63.2 (7.6)	57.8 (13.0)
Blood loss (ml), mean (SD)	97.6 (93.3)	111.4 (72.4)	146.2 (60.3)	117.7 (74.6)
Operative time (min), mean (SD)	57.4 (19.5)	75.3 (21.6)	102.9 (27.8)	78.6 (27.9)
Fluoroscopy time, mean (SD)	189.3 (89.6)	327.9 (130.5)	457.1 (236.7)	330.1 (182.2)
Hospital stay, mean (SD)	0.4 (0.8)	0.9 (1.3)	1.6 (1.9)	1.0 (1.4)

Preoperative and one-year postoperative modified ODI scores by component are shown in Table 3. The mean preoperative ODI score was 49.12%, which improved to 30.71% at one-year follow-up (p<001). There were statistically significant improvements in each ODI category except for standing and traveling.

**Table 3:** Preoperative and postoperative Oswestry Disability Index (ODI) scores

Patient-reported outcomes (mean, SD)	Preoperative ODI	Postoperative ODI	p-value
n	25	17	
Pain	3.56 (1.19)	2.12 (1.65)	0.002
Care	2.20 (1.22)	1.00 (1.17)	0.003
Lifting	3.00 (1.04)	2.24 (1.25)	0.037
Walking	2.56 (1.36)	1.29 (1.10)	0.003
Sitting	1.88 (0.93)	1.12 (1.05)	0.018
Standing	2.36 (1.38)	2.12 (1.76)	0.621
Sleeping	2.12 (1.05)	1.35 (1.54)	0.062
Social	2.52 (1.29)	1.24 (1.44)	0.004
Traveling	1.96 (1.24)	1.35 (1.37)	0.143
Housework	2.40 (1.00)	1.53 (1.01)	0.009
Score	49.12 (14.58)	30.71 (17.87)	0.001

Complications

Complications of this surgery include five cases of nerve root irritation immediately following surgery; two of these cases resolved within 3-6 months of surgery. Three patients had slight nerve root irritation manifesting as mild L5 numbness at last follow-up, with one of these patients being lost to follow-up after four months. None of these cases were clinically limiting. There were no cases of neuropraxia.

## Discussion

Recent advances in surgical techniques and technology have led to the increasing feasibility of minimally invasive methods of spinal treatment. While the literature suggests that minimally invasive spine fusion techniques are able to decrease the morbidity associated with open techniques while achieving similar clinical outcomes, the optimal method of MIS LIF remains an area of debate [15-17].

The L5-S1 spinal level presents unique challenges for minimally invasive interbody fusion. There are currently two common approaches to minimally invasive interbody fusion at the L5-S1 level, anteriorly and posteriorly. Minimally invasive TLIF (MIS-TLIF) and, to a lesser extent, posterior lumbar interbody fusion (PLIF) are common and effective posterior approaches to fusion at all spinal levels. However, the iliac crest and abnormal sagittal angle at the L5-S1 level result in a decreased ability to restore foraminal height and lumbar lordosis when compared to other levels [18-19]. The ALIF is particularly suited to the L5-S1 level due to easier vascular access below the bifurcation of the aorta and inferior vena cava and the ability to place a large, lordotic cage following discectomy [20-21]. However, as an anterior approach, it often requires an access surgeon, and the patient must be repositioned for posterior instrumentation. Furthermore, the anterior approach is associated with comparatively high rates of approach-related morbidity [22]. OLIF-ATP is an alternative anterior approach and allows for the insertion of a large, lordotic cage, sometimes without the utilization of an access surgeon [19]. This approach often requires repositioning of the patient, which can extend the operative time and has a greater risk of vascular, gastrointestinal, and urological complications at the L5-S1 level [23]. While all these approaches are being successfully utilized, they all have notable limitations. Thus, further investigation is necessary to optimize techniques to overcome anatomical difficulties.

Our paper presents an initial proof-of-concept study that demonstrates that the TF-OLLIF has been a safe and effective method of interbody fusion at the L5-S1 level. It is important to note that this adaptation of the OLLIF is not necessary for OLLIF to be used at the L5-S1 level; our previous study reported 152 cases of OLLIF, including the L5-S1 level, of which this cohort comprises a relatively small percentage. However, the TF-OLLIF procedure allows a surgeon to intraoperatively adapt to anatomic barriers, which would otherwise result in reverting to an open technique at this level.

The OLLIF procedure has been shown to result in improved perioperative outcomes compared to both open lumbar fusion surgeries and other minimally invasive techniques [8]. Our previous review of our first 303 OLLIF patients reported a mean single-level operative time of 56.6 minutes, mean single-level EBL of 42.2, and mean single-level LOS of 2.2 days [9]. These numbers are lower than reported open perioperative outcomes and compare favorably to the following predominantly single-level reported mean outcome ranges for MIS techniques: operative time of 104 to 390 minutes, EBL of 51 to 496 ml, LOS of 1.8 to 11 [7]. The spinal fusion procedure is completed through a single 10 mm incision which results in the sparing of paraspinal muscle tissue and avoids many approach-related morbidities associated with dissection and access of other approaches. The conical tip of the interbody allows for the placement of an appropriately sized interbody even with a posterior approach that can increase foraminal space and achieve physiologic decompression. The fusion rate for this procedure has been assessed to be 98.7% by two independent radiologists, and in the largest study of reported cases resulted in an 18-point improvement in ODI score [9].

One of the promising results of this study has been the retention of many of the positive aspects of OLLIF in the TF-OLLIF technique. Similar to OLLIF, TF-OLLIF can be performed through a 10 mm incision and performed through a 10 mm access portal, which reduces iatrogenic trauma to muscle and soft tissue. The ODI improvement of 18.41 compares favorably to previous OLLIF outcomes and other MIS fusion techniques. Estimated blood loss was higher than previously reported OLLIF results for surgeries of one, two, and three levels but comparable to other reported MIS outcomes. Hospital stay was lower than our previous OLLIF results, with the majority of patients who underwent both one-level and two-level surgeries going home the same day.

The operative times reported in this study were exceptional in comparison to most MIS fusion procedures but higher than previously reported OLLIF results. We believe that modification of instrumentation could help us address deficiencies in the technique that currently lengthen the surgery. We have found that the bluntness and width of the 8 mm drill, normally used for discectomy, makes it difficult to quickly drill through the facet prior to discectomy. Advanced Research Medical, the manufacturer of the OLLIF instrument set, is currently developing a system utilizing alternating use of a small and large drill to ameliorate this difficulty.

The main limitation of this study was the fact that it was a retrospective analysis of a cohort of only 29 surgeries performed by a single surgeon. Further study is needed to demonstrate the efficacy of the technique in comparison to other prominent methods of L5-S1 fusion, but the initial results were promising. Another limitation is that TF-OLLIF is performed in conjunction with regular OLLIF levels for all cases except for the seven one-level cases, making it difficult to isolate the impact of TF-OLLIF.

## Conclusions

This was a proof-of-concept study that reported the clinical and perioperative outcomes of the first 29 patients who underwent the transfacet OLLIF procedure. This surgery was initially devised as an adaptation of the OLLIF procedure using OLLIF instruments to approach difficult L5-S1 segments by drilling through facet hypertrophy to access the L5-S1 disc space. This allows a surgeon to achieve interbody placement utilizing OLLIF principles and instruments from T12-S1 even with challenging patient anatomy. Patients in this series were noted to have a similar rate of complications when compared with the traditional OLLIF approach without compromising surgical outcomes. This preliminary data validates the TF-OLLIF procedure and strongly supports the need for a prospective randomized trial.
